# Characteristics of superficial esophageal squamous cell carcinomas undetectable with narrow-band imaging endoscopy

**DOI:** 10.1093/gastro/goab028

**Published:** 2021-08-06

**Authors:** Shingo Ono, Akira Dobashi, Hiroto Furuhashi, Akio Koizumi, Hiroaki Matsui, Yuko Hara, Kazuki Sumiyama

**Affiliations:** Department of Endoscopy, The Jikei University School of Medicine, Tokyo, Japan

**Keywords:** esophageal squamous cell carcinoma, narrow-band imaging, Lugol chromoendoscopy, Lugol-voiding lesions

## Abstract

**Background:**

The detection rate of narrow-band imaging (NBI) for superficial esophageal squamous cell carcinoma (SESCC), including high-grade intraepithelial neoplasia, is significantly higher than that of white-light endoscopy. However, there are SESCCs that are undetectable by NBI but detectable by Lugol chromoendoscopy (LCE) and the characteristics of these SESCCs are still unknown. Thus, this study aimed to clarify the characteristics of SESCC that are undetectable using NBI.

**Methods:**

Patients with current SCC or a history of SCC in the head and neck or in the esophagus were enrolled. The inspection of the esophagus was initiated by NBI, followed by LCE. Biopsies were taken of all suspected SESCC lesions during NBI observation and Lugol-voiding lesions (LVLs) that were irregularly shaped and >5 mm and/or pink in color during LCE observation. The characteristics of SESCC that were undetectable with NBI were statistically analysed.

**Results:**

Overall, 147 lesions in 105 cases were histologically diagnosed as SESCC. Twenty in 15 cases were NBI-undetectable lesions, all of which were macroscopic flat type (0-IIb). The median sizes of the NBI-undetectable lesions and NBI-detectable lesions were both 15 mm (*P *=* *0.47). Multivariate analysis revealed independent factors for NBI-undetectable lesions such as numerous irregularly shaped LVLs (odds ratio [OR]: 4.94, 95% confidence interval [CI]: 1.39–17.5, *P *<* *0.05) and anterior wall position (OR: 4.99, 95% CI: 1.58–15.8, *P *<* *0.05).

**Conclusions:**

The detection of SESCCs with NBI is challenging when lesions are morphologically completely flat, in cases with numerous irregularly shaped LVLs, and if located at the anterior wall.

## Introduction

Esophageal cancer (EC) ranks seventh in terms of incidence and is the sixth leading cause of cancer-related death worldwide [1]. Squamous cell carcinoma (SCC) of the esophagus accounts for >80% of the disease [2]. The prognosis of esophageal SCC (ESCC), especially in advanced lesions, is poor, even after surgical resection, chemotherapy, and radiotherapy [3, 4]. However, the 5-year survival rate after endoscopic submucosal dissection is 99.0% in early-stage ECs without lymph-node metastasis [5]. Therefore, early detection of EC is essential to improve the prognosis.

For decades, the narrow-band imaging (NBI) system has been used with a superficial ESCC (SESCC) diagnosis sensitivity of 82.2%–100% and a diagnostic accuracy of 86.7%–91.2% [6, 7]. However, there are still some SESCCs that are undetectable using NBI, but detectable only using Lugol chromoendoscopy (LCE) even through careful esophageal examination by expert endoscopists. LCE is usually performed after NBI observation for high-risk patients of SESCC, in order not to overlook those lesions. However, the characteristics of SESCC that are undetectable by NBI remain unknown. This study aimed to clarify the characteristics of SESCC that are undetectable through NBI. Since the sensitivity of LCE is higher than that of NBI [8], we chose LCE for the gold standard in this study. We retrospectively analysed the SESCC diagnosed in high-risk patients who underwent both NBI and LCE on the same day.

## Materials and methods

### Study design and population

This study served as a post-hoc analysis of the previous prospective studies conducted at the Jikei University School of Medicine (Tokyo, Japan) [6, 9]. These prospective studies recruited high-risk patients with ESCC from January 2009 to October 2015 to determine the effectiveness of NBI in diagnosing SESCC. The present retrospective study was approved by the institutional review board of the Jikei University School of Medicine 30–408 (9429).

### Inclusion and exclusion criteria

The inclusion criteria were as follows: (i) patients with current SCC or a history of SCC in the head and neck or the esophagus; (ii) patients who underwent both NBI-magnifying endoscopy (NBI-ME) and LCE on the same day for screening or surveillance of EC; (iii) patients whose endoscopic information and previous records of esophageal endoscopic findings were blinded owing to the prospective trial.

The exclusion criteria were as follows: (i) patients who had a prior history of radiotherapy for EC; (ii) patients who received systemic chemotherapy; (iii) patients with synchronous advanced esophageal carcinoma; (iv) patients in whom endoscopy was unable to pass through because of stenosis of the esophagus; (v) patients without a pathological diagnosis owing to the difficulties in discontinuing antithrombotic medication.

### Endoscopic procedure

A conventional magnifying endoscope (GIF-H260Z; Olympus Corporation, Tokyo, Japan) or Dual-Focus endoscope (GIF-HQ290; Olympus Corporation, Tokyo, Japan) was used in this study. We used a black rubber distal attachment (MB-46 [Olympus, Tokyo, Japan] for GIF-H260Z or MAJ-1989 [Olympus, Tokyo, Japan] for GIF-HQ290) on the tip of the endoscope to maintain the focal distance between the tip of the scope and it facilitated precise focusing during the magnification observation to inspect the esophagus in all eligible patients. All endoscopic examinations were performed under conscious sedation using intravenous flunitrazepam (0.2–0.8 mg, Rohypnol; Chugai Pharmaceutical, Tokyo, Japan) and pethidine hydrochloride (17.5–35 mg, Pethidine; Takeda Pharmaceutical, Osaka, Japan). Patients were initially examined by NBI. After observation of the stomach and duodenum, LCE was performed using 1.5% iodine solution. All procedures were performed by expert endoscopists, who were Japan Gastroenterological Endoscopy Society board-certified instructors with NBI examination experience of >1,000 cases.

### Biopsy protocol

Under NBI with standard mode (non-magnification mode), well-demarcated brownish areas >5 mm in diameter or depressed/elevated lesions were detected as suspected SESCC lesions. The NBI-ME (including near focus mode) was performed to distinguish SESCC from low-grade intraepithelial neoplasia (LGIN) or benign lesions based on the NBI magnification findings [10, 11]. During LCE, irregularly shaped Lugol-voiding lesions (LVLs) with diameter >5 mm and/or pink in color were further detected as suspected SESCC lesions. The shape of the LVLs was evaluated according to the previous report [12]. A lesion with a positive pink color sign was biopsied as the first priority and a larger size was the next priority. Targeted biopsies were obtained from all suspected SESCC lesions that were detected under non-magnified NBI or LCE. The number of biopsies under LCE was limited to up to five biopsies for each case.

### Pathologic diagnosis

Histologic diagnosis was classified as no neoplasia, indefinite for neoplasia, LGIN, high-grade intraepithelial neoplasia (HGIN), or SCC [13, 14]. The depth of the tumor invasion was defined as epithelium including HGIN, lamina propria mucosae (LPM), muscularis mucosa (MM), and submucosae (SM). A submucosal SESCC is subclassified as SM1 (invading to a depth of <200 µm from the MM) or SM2 (extending >200 µm). The SESCC was defined as HGIN and/or SCC with invasion confined to the submucosa. Lesions diagnosed as no neoplasia, indefinite for neoplasia, or LGIN were treated as non-SESCC. If the histologic results of a lesion differed between the biopsied and resected specimens, the worst malignant histology was used.

### Definition of NBI-undetectable SESCCs

If an endoscopist identified the new lesion as LVL under LCE and the histology of the lesion revealed HGIN/SCC, then the lesion was classified as NBI-undetected SESCC. Once the lesion was detected by NBI, it was regarded as an NBI-detectable lesion even though the diagnostic system including Inoue’s classification [11] or dyad criteria [9] evaluated the lesion as non-SESCC. After spraying iodine, it is impossible to look back at the NBI observation in the iodine-stained esophagus and conduct careful observation under NBI-ME. Some NBI-undetected SESCCs could be observed the next day under NBI-ME before endoscopic resection. We reviewed those images and evaluated the color changes under white-light imaging (WLI), the presence of well-demarcated brownish areas under NBI, and six NBI magnification findings [9].

### Outcomes

The primary endpoint was the determination of the characteristics of SESCC that were undetected using NBI. We analysed the relationship between NBI-detectable and NBI-undetectable groups (i.e. detected only by LCE) for the following variables: macroscopic type, tumor size, lesion location (cervical esophagus [Ce], upper thoracic esophagus [Ut], middle thoracic esophagus [Mt], lower thoracic esophagus <, and abdominal esophagus [Ae]), position, grades of LVL pattern, the existence of synchronous SESCCs, and tumor depth. The lesion location was determined by the center of the lesion. The LVL pattern was classified according to Muto’s classification as follows: A, absence of LVLs; B, several (≤10) small LVLs; C, many (>10) small LVLs; and D, numerous irregularly shaped multiform LVLs [15]. A synchronous lesion was defined as histological SESCCs detected in more than two lesions during the observation. A lesion located from 9 o'clock via 12 o'clock to 3 o'clock when the spine was located at a 6 o'clock position was defined as an anterior lesion. The other lesions were classified as non-anterior lesions.

### Statistical analysis

The student *t*-test or Mann–Whitney *U* test was used to compare continuous variables and Fisher’s exact test or a chi-square test was used to compare the proportions of categorical variables. Risk factors for NBI-undetectable SESCCs were analysed using a logistic-regression model. Factors that may influence non-NBI detection were analysed by univariate logistic-regression analysis, and factors with a *p*-value of <0.05 were analysed by multiple logistic regression under the forced-entry method. A *p*-value of <0.05 was considered statistically significant. All analyses were performed using the statistical program R.

## Results

Of the 316 patients who met the criteria, a total of 105 consecutive patients with 147 lesions were histologically diagnosed with SESCCs (Table 1). The median age of the patients was 69 years and males were predominant (96.2%). Overall, 227 biopsies were obtained from 105 patients and 64.8% (147/227) were histologically HGIN or SCC. Under NBI, 160 lesions were detected, of which 127 were diagnosed as SESCC. After spraying iodine, 67 LVLs were newly identified as suspected SESCC lesions, of which 20 were histologically SESCCs (Figure 2). In a per-patient analysis, an average of 2 (range, 1–4) biopsies were added during LCE, and SESCCs were newly diagnosed in 15 of 56 patients. The number of biopsies was significantly higher in patients with NBI-undetectable lesions than in patients with only NBI-detectable lesions (3 vs 2, *P *<* *0.05).

**Figure 1.**
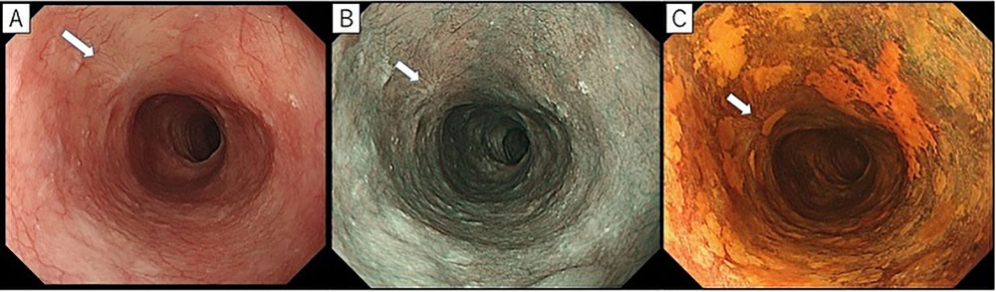
A representative case of NBI-undetectable superficial esophageal squamous cell carcinoma. (A) The lesion was not detected under WLE in the esophagus. ESD scar (white arrow) is noticed at the 10 o’clock position. (B) On non-magnifying NBI endoscopy, the brownish area was not clearly identified. **(**C) The LVL appeared on the anterior wall by LCE with multiple irregularly shaped LVLs in the background mucosa. NBI, narrow-band imaging; WLE, white-light endoscopy; ESD, endoscopic submucosal dissection; LCE, Lugol chromoendoscopy; LVL, Lugol-voiding lesion.

**Figure 2.**
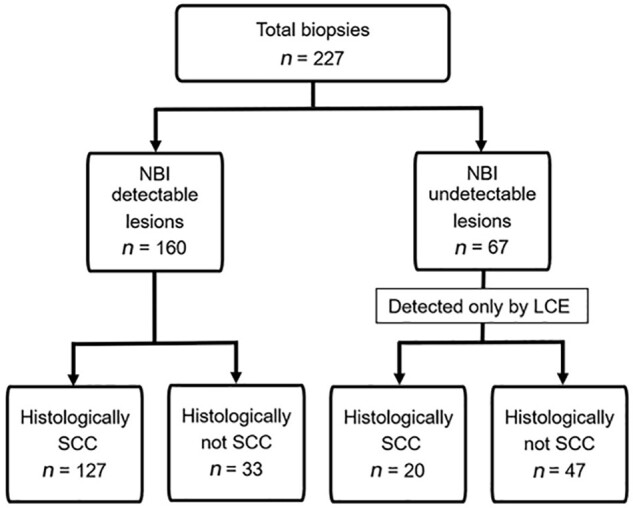
Flowchart of total biopsies performed of NBI-detectable and undetectable lesions, followed by LCE. NBI, narrow-band imaging; LCE, Lugol chromoendoscopy; SCC, squamous cell carcinoma.

**Table 1.** Demographics of patients and features of lesions

Characteristic	Value
Patients	*n *=* *105
Age, years, median (range)	69 (50–88)
Men, *n* (%)	101 (96.2)
Lesions	*n *=* *147
Pathology, *n* (%)	
Squamous cell carcinoma	108 (73.5)
HGIN	39 (26.5)
Diameter, mm, median (range)	15 (2–110)
Macroscopic tumor type, *n* (%)	
0-I	7 (4.8)
0-IIa	10 (6.8)
0-IIb	97 (66.0)
0-IIc	31 (21.1)
0-III	2 (1.4)
Depth of tumor invasion, *n* (%)	
T1a + HGIN	116 (78.9)
T1b	14 (9.5)
Unknown^a^	17 (11.6)

HGIN, high-grade intraepithelial neoplasia.

a Lesions were not resected (e.g. treated by chemoradiotherapy).

Of the 147 SESCCs, the treatment was as follows: endoscopic resection (*n *=* *86), surgical resection (*n *=* *24), chemotherapy and/or radiotherapy (*n *=* *12), ablation with argon plasma coagulation (*n *=* *3), biopsy removal (*n *=* *8), and others (*n *=* *14) were observed without treatment. Of the 147 SESCC cases, 116 were intramucosal carcinomas (including HGIN), 14 were submucosal carcinomas, and 17 were unknown.

The characteristics of NBI-undetectable lesions are summarized in Table 2. Twenty SESCCs in 15 patients were NBI-undetectable lesions. The median sizes of the NBI-undetectable and NBI-detectable lesions were both 15 mm (*P *=* *0.47). The macroscopic type in all NBI-undetectable lesions was completely flat (0-IIb). Seven lesions were histologically HGIN and 13 lesions were SCCs (T1a-LPM, *n *=* *7; T1a-MM, *n *=* *2; T1b-SM, *n *=* *0; invasion depth unknown, *n *=* *4) in NBI-undetectable lesions. Univariate analysis revealed that the use of H260Z endoscope (22.9% vs 5.2%), numerous irregularly shaped LVLs (22.5% vs 5.3%), synchronous SESCC (21.1% vs 6.6%), and lesions located at the anterior wall (23.0% vs 7.0%) were significantly associated with an increased rate of NBI-undetectable lesions (all *P *<* *0.05). There were no significant differences in age, sex, lesion location, median tumor size, or histological diagnosis between the two groups. Multivariate analysis revealed numerous irregularly shaped LVLs (odds ratio [OR], 4.94; 95% confidence interval [CI], 1.39–17.5; *P *<* *0.05) and position at the anterior wall (OR, 4.99; 95% CI, 1.58–15.8; *P *<* *0.05) as independent factors for NBI-undetectable lesions (Table 3).

**Table 2.** Univariate analysis for NBI-undetectable lesions

Characteristic	No. of lesions	NBI-undetectable (*n *=* *20)	NBI-detectable (*n *=* *127)	*P*-value
Endoscope system, *n* (%)				<0.05
H260Z	70	16 (22.9)	54 (77.1)	
HQ290	77	4 (5.2)	73 (94.8)	
Size, mm, median (range)	147	15 (6–35)	15 (2–110)	0.5
Location, *n* (%)				0.8
Ce+Ut	42	6 (14.3)	36 (85.7)	
Mt	78	11 (14.1)	67 (85.9)	
Lt+Ae	27	3 (11.1)	24 (88.9)	
Position, *n* (%)				<0.05
Anterior wall	61	14 (23.0)	47 (77.0)	
Others	86	6 (7.0)	80 (93.0)	
Macroscopic type, *n* (%)				<0.05
0-IIb	97	20 (20.6)	77 (79.4)	
Others	50	0 (0)	50 (100.0)	
Pathology, *n* (%)				0.4
HGIN	39	7 (17.9)	32 (82.1)	
SCC	108	13 (12.0)	95 (88.0)	
Numerous irregularly shaped LVLs, *n* (%)				<0.05
Yes	71	16 (22.5)	55 (77.5)	
No	76	4 (5.3)	72 (94.7)	
Synchronous SESCCs, *n* (%)				<0.05
Yes	71	15 (21.1)	56 (78.9)	
No	76	5 (6.6)	71 (93.4)	

NBI, narrow-band imaging; Ce, cervical esophagus; Ut, upper thoracic esophagus; Mt, middle thoracic esophagus; Lt, lower thoracic esophagus; Ae, abdominal esophagus; HGIN, high-grade intraepithelial neoplasia; LVLs, Lugol-voiding lesions; SESCC, superficial esophageal squamous cell carcinoma.

**Table 3.** Multivariate analysis for NBI-undetectable vs NBI-detectable lesions

Factor	OR (95% CI)	*P*-value
Numerous irregularly shaped LVLs	4.94 (1.39–17.5)	<0.05
Position, anterior wall	4.99 (1.58–15.8)	<0.05
Synchronous SESCCs	2.86 (0.85–9.60)	0.09
Macroscopic type, 0-IIb^a^	–	–

NBI, narrow-band imaging; OR, odds ratio; CI; confidence interval; LVLs, Lugol-voiding lesions; SESCC, superficial esophageal squamous cell carcinoma.

a NBI-undetectable lesions were all 0-IIb and macroscopic type was omitted because of collinearity.

Twelve of 20 NBI-undetectable lesions could be observed with NBI magnification before endoscopic resection (Supplementary Figures). The result of the characteristics of NBI magnified findings are shown in Table 4. Most of the lesions did not show changes in color under WLI (83.3%, 10/12) and did not show a well-demarcated brownish area under NBI (83.3%, 10/12). In addition, under NBI-ME, those lesions had slight change of intrapapillary capillary loop (IPCL) (i.e. proliferation or dilatation) and did not meet Inoue’s criteria [11].

**Table 4.** The evaluation of the intrapapillary capillary loop in NBI-magnifying images for NBI-undetectable SESCCs

Finding	*N* = 12^a^
Intervascular background coloration	4 (33.3)
Proliferation	7 (58.3)
Dilation	11 (91.7)
Tortuosity	2 (16.7)
Change in caliber	2 (16.7)
Various shapes	3 (25.0)

NBI, narrow-band imaging; SESCC, superficial esophageal squamous cell carcinoma.

a NBI-magnifying endoscopy was performed for 12 NBI-undetectable SESCCs on another day before endoscopic resection.

## Discussion

This was the first report to clarify the characteristics of SESCCs that are undetectable using NBI. NBI-undetectable SESCCs were all macroscopically completely flat type and the statistical analysis revealed that numerous irregularly shaped LVLs and lesions located at the anterior wall are independent risk factors for undetectable SESCCs by NBI even though high-definition magnifying endoscopy was used by expert endoscopists.

The NBI is an innovative image enhancement technology that significantly increased the detection rate of SESCC compared to WLI [16]. Image-enhanced endoscopy such as NBI can enhance the slight reddish color change and allows easier visualization of images with higher contrast than conventional WLI [17]. In WLI, completely flat type (0-IIb) lesions are the most challenging to detect for SESCC because the difference in redness is the only key feature. The completely flat type is more common in ESCC compared to other gastrointestinal cancers (i.e. early gastric cancer and early colorectal cancer) and the color difference is very slight in some lesions. Thus, detecting SESCCs requires skill and experience, and the SESCC detection rate by WLI remains at 60% even for experts .

**Figure 3.**
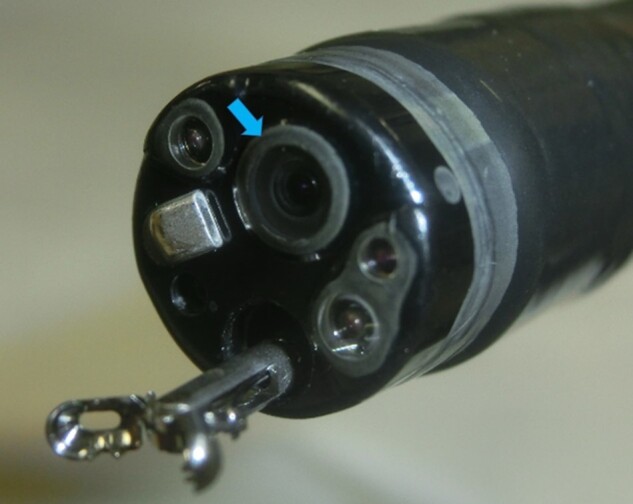
A picture of the tip of the endoscope. The blue arrow indicates the objective lens located at the upper side.

In the NBI system, the light range during observation is narrow and blood hemoglobin easily absorbs light of specific blue and green wavelengths (415 nm, 540 nm) that is used to enhance superficial capillaries for lesion detection. Although advantageous, the light intensity is lower compared to WLI. Unless the light hits the target gastrointestinal wall vertically, the darker light often makes it challenging to detect SESCC lesions as a well-demarcated brownish area. A further disadvantage of darker light is difficulty in observing the distal area [18]. The number of NBI-undetectable lesions with the HQ290 system in this study was smaller than that of the H260Z system. This may be because the brightness of NBI with the HQ290 system was improved compared to that of the H260Z system. It is often difficult, even for expert endoscopists, to observe the anterior wall of the esophagus due to its tangential view, making appropriate distance challenging. This might be explained by the position of the objective lens, which is at the upper side of the tip of the endoscope (Figure 3) and tends to be closer to the anterior wall of the esophagus. In addition, the anterior wall of the esophagus has external displacement caused by left atrium and left main bronchus, as well as fluctuations following heartbeat and respiration. These factors may influence lesion detection at the anterior wall of the esophagus by NBI, especially for completely flat lesions.

The advantage of LCE is the strong contrast between stained (i.e. background mucosa) and unstained areas (i.e. SCC or intraepithelial neoplasia). Since the light intensity is comparable to that of WLI, the color difference may not be strongly affected by the angle or distance between the endoscope and the esophageal wall. Therefore, the lesion position may not be influenced during LCE observation. Considering that the anterior wall is a risk factor for NBI-undetectable lesions, rotating the endoscope by 180° and bringing the anterior wall down during observation may reduce the negligence of SESCCs.

Recognition of the changes in IPCLs is essential for identifying SESCC by NBI observation. However, IPCLs in the background mucosa are also affected for some reasons. It has been reported that IPCL changes in proliferation and elongation are signs of inflammation in the esophagus [19]. In EC with inflammation in the background, it is known that the demarcation line of the lesions is more difficult to recognize in NBI than in LCE because of the additional changes in IPCL specific to inflammation that make the neoplastic lesions more complex. As previously reported, multiple foci of dilated vessels in NBI observation may be a new indicator of multiple LVLs [20]. The esophagus of patients with numerous irregularly shaped LVLs may have a unique IPCL change due to chronic inflammation associated with smoking and alcohol consumption. This further complicates the finding of neoplastic lesions by NBI observation. In LCE observation, less atypical changes such as LGIN may be recognized as a clear LVL, yet not clearly identified in NBI observation. As previously reported, the vascular structure of EC changes during tumor progression [21] and the degree of irregularity is correlated with tumor invasion in SESCCs. However, some SESCCs have mild irregularities in the superficial microvasculature.

The tumor depth of NBI-undetectable lesions was pathologically limited within the MM and no lesions invaded the submucosa in this study. Therefore, there were no advanced stage lesions in NBI-undetectable lesions that might have affected prognosis. Two SESCCs invaded the MM wherein one of these lesions was a 10-mm-sized 0-IIb lesion on the anterior wall of the Ce. This patient had stage 4 oropharyngeal cancer. The other patient had a 20-mm-sized 0-IIb lesion on the anterior wall of the Lt. This patient had a synchronous lesion, a 19-mm-sized 0-IIc + IIa lesion located on the Mt, and the final pathology showed submucosal invasion (800 μm) with vascular invasion. According to these results, NBI-undetectable SESCCs were not the worst lesions and might not have affected the patient’s prognosis. However, depending on the timing of the discovery and the patient’s co-morbidities, prognosis may have been affected.

The NBI remains the primary diagnostic tool for SESCC due to its high sensitivity, diagnostic accuracy, and convenience, whereas LCE may be useful for detecting additional lesions in high-risk patients with EC. Our results suggest that the strategy for reliable screening and surveillance endoscopy for high-risk patients with ESCC is as follows. First, the anterior wall should be carefully observed with a rotating endoscope using peristalsis. Second, if numerous LVLs have been discovered in previous endoscopy or ESCCs were found during NBI observation, LCE should be considered. These strategies may ensure effective LCE and reduce NBI-undetectable SESCCs.

Our study had several limitations. First, this was a retrospective single-center study. The participants were limited to high-risk patients only, and many patients had multiple lesions. It is unclear whether these results can be applied to low-risk patients. Second, all endoscopic procedures were limited to experts in the field; thus, the detection data may fluctuate compared to non-experts. Third, the endoscopic light source device and endoscope (GIF-H260Z, GIF-HQ290; Olympus) were not unified in this study.

In conclusion, the detection of SESCCs with NBI is challenging when they are morphologically completely flat, in cases with numerous irregularly shaped LVLs, or those located at the anterior wall. Thus, LCE should be considered to prevent overlooking SESCC in patients with numerous LVLs observed in previous observations or ESCCs found during NBI observation.

## Supplementary Data

Supplementary data is available at *Gastroenterology Report* online .

## Author’s Contributions

S.O. and A.D. conceived of and designed the project and revised the manuscript. H.M., A.K., and Y.H. collected the data. H.F. analysed the data. K.S. reviewed and edited the manuscript. All authors read and approved the final manuscript .

## Funding

None.

## Supplementary Material

goab028_Supplementary_Data
