# Supplementary figures and images for: Characteristics of superficial esophageal squamous cell carcinomas undetectable with narrow-band imaging endoscopy
Source: Gastroenterol Rep (Oxf). 2021 Aug 6;9(5):402–7. doi: 10.1093/gastro/goab028 (PMC8560036; doi:10.1093/gastro/goab028)

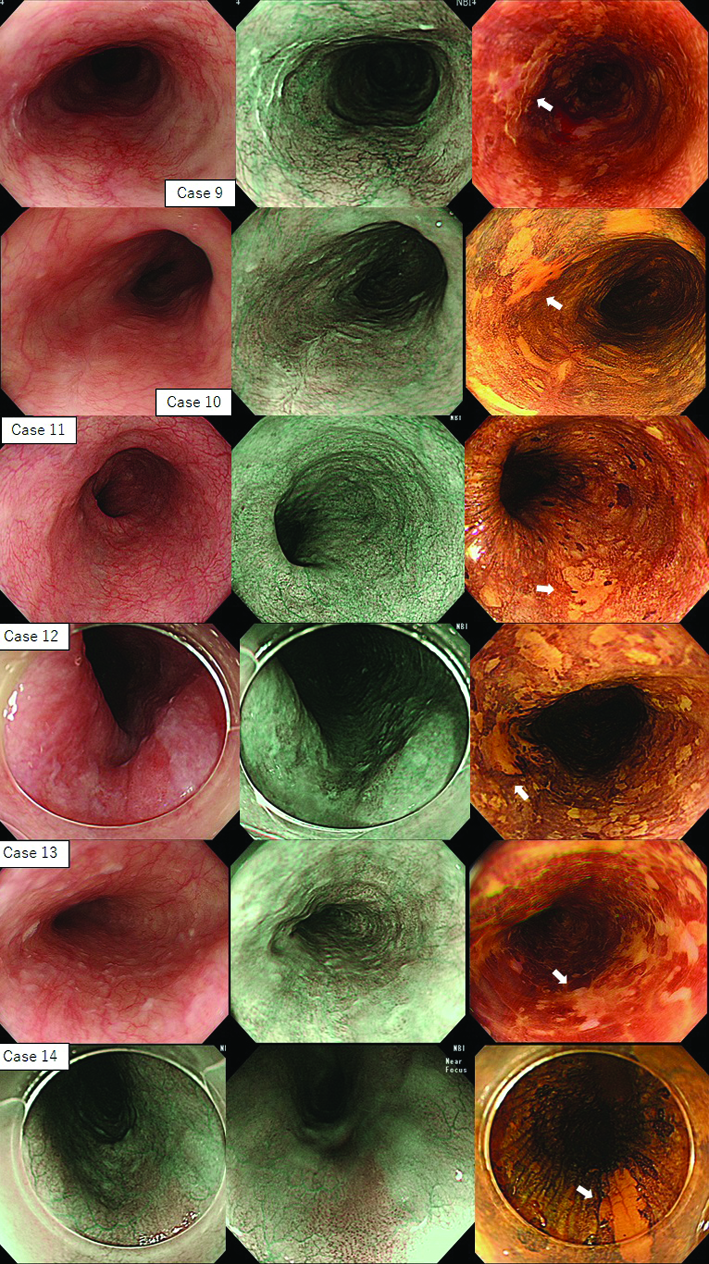

Supplement: goab028_Supplementary_Data [file goab028_supplementary_data.zip › goab028-suppl-data/Sup 2.tif]

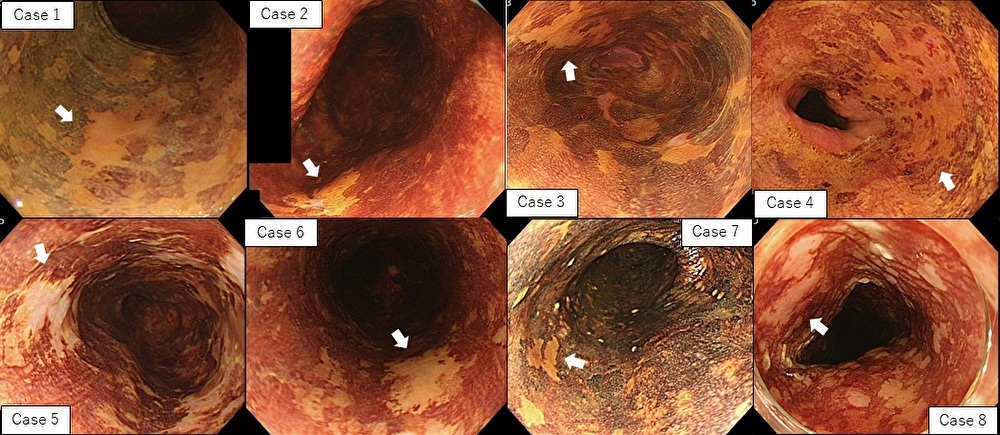

Supplement: goab028_Supplementary_Data [file goab028_supplementary_data.zip › goab028-suppl-data/Sup.1.jpg]

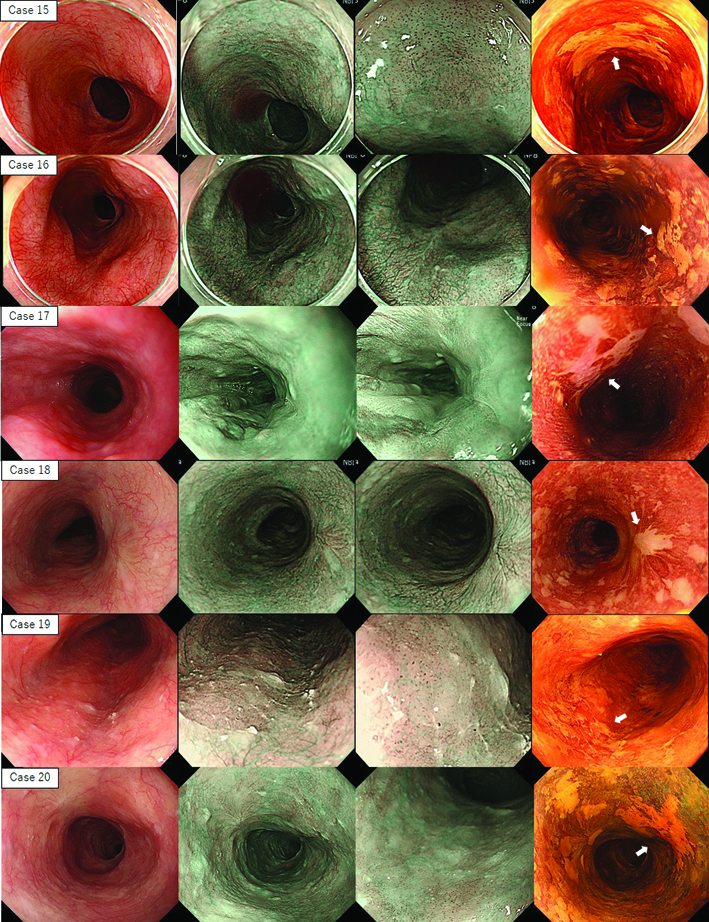

Supplement: goab028_Supplementary_Data [file goab028_supplementary_data.zip › goab028-suppl-data/Sup.3.tif]
